# Numerical Optimization of the Blank Dimensions in Tube Hydroforming Using Line-Search and Bisection Methods

**DOI:** 10.3390/ma13040945

**Published:** 2020-02-20

**Authors:** Antonio Fiorentino, Paola Serena Ginestra, Aldo Attanasio, Elisabetta Ceretti

**Affiliations:** Depth. of Mechanical and Industrial Engineering, University of Brescia (Italy), Via Branze 38, 25123 Brescia, Italy; paola.ginestra@unibs.it (P.S.G.); aldo.attanasio@unibs.it (A.A.); elisabetta.ceretti@unibs.it (E.C.)

**Keywords:** tube hydroforming, AISI 316L, optimization, line–search method, bisection method

## Abstract

The change from a consolidated manufacturing practice to a new solution is often a complex problem because of the operative limits of technologies and the strict constraints of industrial parts. Moreover, the new process must reflect or enhance the characteristics of the product and, overall, it must be more competitive in performances and costs. Accordingly, the development of a new process is a multilevel and multivariate problem that requires a systematic and hierarchical approach. The present paper focuses on the development of a Tube Hydroforming process capable to replace the current practice for production of T-Joint parts made of AISI 316L for the water pipes market. In particular, the problem must withstand many process and product constraints. Therefore, it was split in three steps focused on specific aspects of the process: identification of process parameters and configuration, numerical optimization of the blank tube dimensions (length and thickness), experimental tests and final improvements. In particular, two numerical methods were implemented in the optimization step: the line–search method to approach to the optimum point and Bisection method to refine the search. These approaches allowed us to identify the optimum process configuration and, in particular, the optimal dimensions of the blank tube that allows one to achieve the product requirements with the minimum cost of material.

## 1. Introduction

The change from a consolidated manufacturing process to a new and innovative solution is often a complex problem under two main aspects, process and part feasibilities. In fact, each process is characterized by its own parameters, operating limits and constraints that can influence or compromise the part utilization. Moreover, industrial parts must respect strict constraints since they have to accomplish international standards, regulations and customer’s requirements. Finally, the new product must reflect or enhance the characteristics of the previous one and, overall, it must be more competitive in performances and costs. Accordingly, the development of a new process is a multilevel and multivariate problem that can be faced by establishing a hierarchical investigation so to reduce the number of considered variables.

The present paper focuses on the production of T-Joint parts made in AISI 316L for the water pipes market fabricated by a company with traditional technologies. In fact, the current production process consists of a multiphase process that mainly consists in tube preforming, cutting, welding of the third branch and beads grinding. In order to enhance the product, the company chose to investigate the possibility of replacing the current manufacturing process with Tube Hydroforming (THF) [1]. In THF, a blank tube is placed in hollow dies that are shaped as the desired geometry of the part. Then, the tube edges are sealed by two punches, one of which is hollow and allows one to fill the tube with liquid. After sealing, the pressure of the liquid is raised and the punches compress the tube along its axis. In particular, the liquid acts like a flexible punch that expands the tube from the inside and the punches feed the material in the expansion zones [2]. In the case of T-joint productions, a third punch (namely counter-punch) can sustain the tube during the expansion along the third branch to prevent the tube bursting. Pressure, punch strokes and counter-force curves are the most important process parameters for the part feasibility [1] since they influence the strain paths and gradients that can lead to wrinkle formation or necking and the subsequent bursting of the tube [3]. Moreover, the contact between tube and dies plays a significant role in the process. In fact, friction influences the material flow in the dies and, in particular, it can reduce the amount of material that gets through the expansion zones [4] or it can unbalance the flow when it is not uniform [5,6]. The advantages of THF are the lower assembly time and cost and the higher complexity of the part with respect to traditional tube forming processes [7]. Moreover, the whole blank undergoes plastic deformation, therefore the hardening increases the material mechanical properties [7,8]. Finally, the part weight and dimensional accuracy are enhanced [1,2,7]. For its characteristics, THF is suitable for aeronautic [9], automotive [10,11] and pipe fields, mono [12] and bilayered [13] ones, as in the production of tubular components for fluids delivery, aero-engine, engine cradles, anti-intrusion bars, radiator supports or nozzle production.

The aim of this work is to develop a Tube Hydroforming process capable to replace the current tube cutting and welding production system of the company [7]. In particular, the present approach must withstand many constraints on the material, commercial tube diameter, height expansion and minimum thickness of the part protrusion. Moreover, since the cost of the material is considerable, the amount of material involved significantly affects the cost of the product. Being a complex issue, it was divided in three steps focused on specific aspects of the process: identification of process parameters and tool configuration, numerical optimization of the blank tube dimensions, experimental tests and final process improvement. The first step was conducted using the Finite Element Method (FEM) to identify and to solve the critical aspects of the process. The second step aimed at minimizing the material involved (length and thickness of the blank tube) meanwhile achieving the product requirements. In particular, two numerical optimization methods were adopted, the line–search method [14] to approach to the optimum and the bisection method [15] to refine the search. In the third step, the results of the optimization were experimentally tested and the final adjustments were introduced in the process. Each step optimized a part of the main problem and lead to a process configuration that was used as a start of the following step. For confidentiality reasons, the paper discloses the data of the process in aggregated or normalized forms.

The step-by-step procedure was successfully tested on two sizes of T-Joint and allowed us to develop a leaner manufacturing process that respects the requirements of the products and enhances their characteristic. Moreover, the results showed the capability of the method in optimizing a multivariate THF process.

## 2. Materials and Methods

### 2.1. Part Geometries

**Figure 1:**
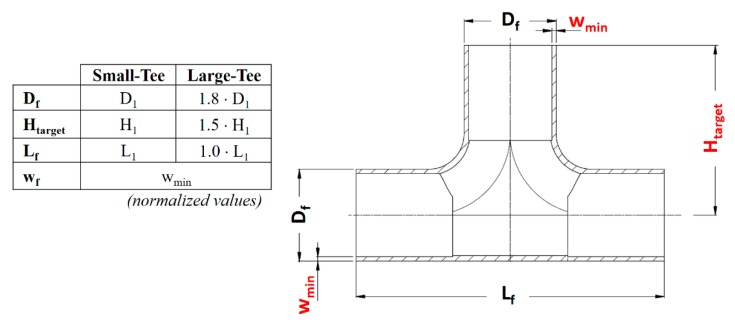
Geometries of the T-Joints to be manufactured by Tube Hydroforming (THF).

The parts considered in this study were two sizes of T-joints (Figure 1), which were produced by a company (Raccorderie Metalliche S.p.A. Campitello di Marcaria, Italy) leader in the pipe production field. The current production process consists of partial hydroforming that generates a little bulge in the tube, cutting of the dome, welding of the third branch and, finally, the three branches are bored to the desired diameter. The interest of the company is to fully form the third branch through hydroforming so to reduce lead time and cost of the product.

Therefore, the hydroforming process was studied focusing on two specific T-joints, namely the Small-Tee and Large-Tee (Figure 1). The parts have similar critical features, in particular they require a:

- Long bulge height (H_target_) compared to the tube diameter (H_target_/D_f_ equal to 1.9/1 and 1.5/1 respectively),
- Minimum wall thickness (w_min_),
- Outer diameter corresponding to commercial blank tubes (available and suitable for the Company),
- AISI 316L material to comply with drinking water standards.

The different tube dimensions (final diameter-D_f_, final length-L_f_ and final wall thickness-w_f_) were chosen to test the optimization method at two product scales, large and small, where the target deformation is respectively easier and more difficult to achieve.

### 2.2. FEM Model

**Figure 2:**
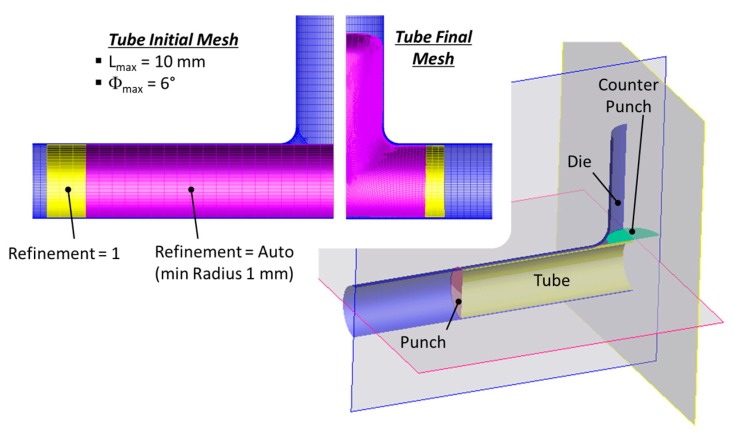
Finite Element Model (FEM) and tube meshing strategy.

**Figure 3:**
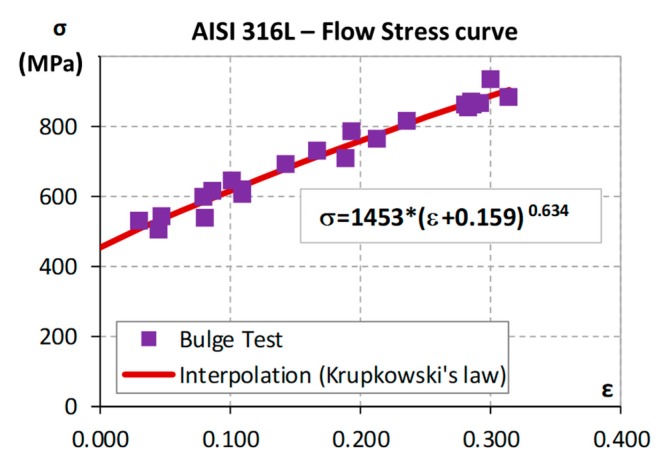
AISI 316L flow stress curve.

The FEM model used in the research was developed in PamStam2G code (Figure 2 and Table 1). In particular, the model has two symmetry planes, the flow stress curve of the material was experimentally estimated using the bulge test [16,17] and results were interpolated adopting the Krupkowski’s law (Figure 3), the tube–die interface was lubricated with solid film and the coefficient of friction was estimated using large scale pin-on-disk equipment reported in [18]. Moreover, a shell mesh was used for all bodies and a specific meshing strategy was adopted for the tube to avoid model instabilities. In fact, the edges of the tube are strongly compressed along its axis by the punches and the thickness strongly increases [19]. Therefore, wide mesh elements are required so to keep the mesh size-thickness ratio over 75% (limit value for using shell elements with the FEM code [20]). Moreover, the tube edges are not remeshed to keep wide the elements close to the punch. On the contrary, in the expansion area of the tube, the axial compression is coupled with a high radial stretching, which leads to long but thin elements that are too distorted to be computed. To avoid these instabilities, a local refinement strategy was adopted (Figure 2). In particular, the core mesh was refined during the simulation to remesh the distorted elements.

## 3. Process Optimization

The optimization of a hydroforming process requires the identification of the optimal process parameters curves (liquid pressure and stroke of the punches), tools geometries (die and counterpunch) and blank tube dimensions (diameter, thickness and length). Moreover, the considered parts have strong constraints on material and final geometry. Therefore, a large number of variables and data have to be considered in the optimization procedure. In order to simplify the approach, the optimization was split in three steps, each focusing on specific aspects of the process. In particular:

- Step 1—process parameter curves. In this step, a FEM model was used to preliminary investigate the process and to identify which configurations enhance the feasibility of the part. At the end of the step, proper process parameters (pressure, punch strokes and counter-force curves) were identified and kept constant in the following steps so to focus the further analysis on less variables. Moreover, the limits where the model is stable (stability domain) were identified in terms of maximum and minimum tube dimensions (length and thickness).
- Step 2—numerical optimization. After the reduction of the number of free variables through the identification of the process curves, this step adopted a systematic approach based on numerical optimization techniques with the aim of identifying the dimensions of the tube (length and thickness, i.e., minimum amount of material) that guarantee the part requirements in terms of final height and thickness (H_target_ and w_min_). Their numerical optimization was constrained by the stability domain of the FEM.
- Step 3—experimental tests and final adjustments. The previous steps identified a process configuration, which is close to the optimum. Therefore, it was used as a starting point for an experimental campaign that tuned the industrial process. At the end of this step, the optimized process parameters for the production of the two parts were identified.

### 3.1. FEM Model

The FEM model was used to preliminary investigate the process so to evaluate which are its critical aspects in terms of the part feasibility.

**Figure 4:**
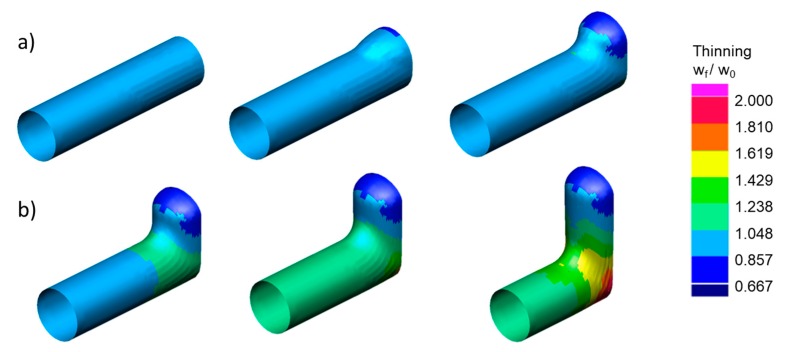
Small-Tee. Thinning at different instants of the hydroforming process. (**a**) initial deformation phase; (**b**) final deformation phase.

Figure 4 reports the thickness of the part during the forming process. In particular, it shows that the tube thinned on the dome of the expansion while it thickened on the sides. Therefore, the critical feature of the part when hydroformed was the height of the bulge whose thickness has to be higher than w_min_ (i.e., H(w_min_) ≥ H_target_). Moreover, dome thinning occurred at the beginning of the process (Figure 4a) and then it settled (Figure 4b). Therefore, H(w_min_) mainly depended on the mechanism of the initial deformation.

**Figure 5:**
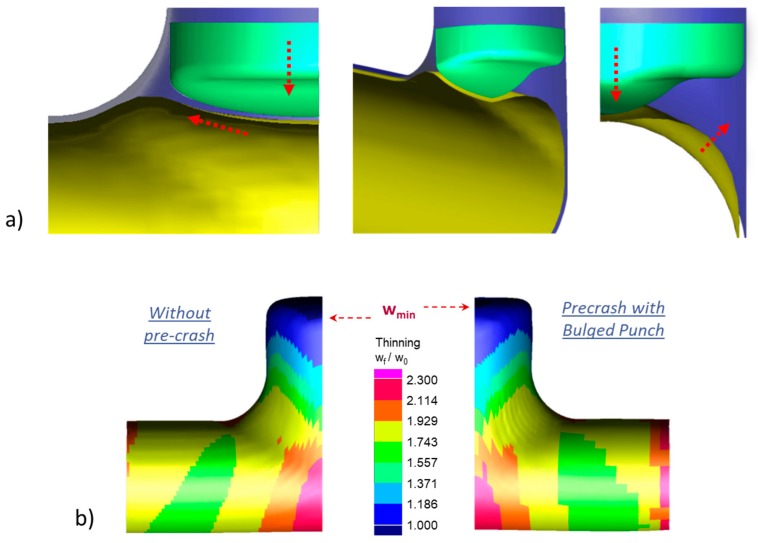
Precrash of the tube with bulged counter-punch. Effects on the (**a**) material flow (**b**) and thinning.

Accordingly, different simulations were run to determine the process configuration that minimizes the thinning at the first stages of THF. Initially, the influence of pressure, punch velocity and counter-punch force were investigated. Results suggested to use the minimum pressure at the beginning of the process while it was irrelevant afterwards (Table 2). Other process curves (punch velocity and counter-punch force) did not significantly influence the thinning. Finally, a precrash with a bulged counter-punch was simulated (Figure 5a) to evaluate whether the part formability is enhanced by preforming the bulge area [21,22]. Results showed that the precrash of the tube increased H(w_min_) almost to the top of the dome (Figure 5b).

Once identified the process parameter curves, the stability of the FEM model was tested. In particular, different combinations of the initial tube length (L) and thickness (w) were used to evaluate where numerical and process instabilities occur. Results showed that too thick or long tubes led to FEM instabilities. Moreover, too thin or short tubes did not allow us to accomplish the part requirements. By merging these constraints, a feasibility domain of the tube dimensions was identified (Table 2).

At the end of this step, the requirement of the part H(w_min_) ≥ H_target_ was not satisfied, but a process configuration that allowed us to maximize the formability and the stability domain of the FEM model of the part was identified (Table 2).

### 3.2. Tube Dimensions

The previous step showed that the part had a thickness that was globally higher than w_min_ (Figure 5b). In particular, the punches compressed the material that partially flowed through the third branch of the part and the rest accumulated on the other two lateral branches. This led to an excess of material and, consequently, to a high cost of the part. Moreover, the constraint H(w_min_) ≥ H_target_ was not accomplished by using only the optimized process curves (Table 2).

H(w_min_) can be enhanced by increasing the initial tube length (L), which means more material to use and, hence, more exceeding material on the sides of the part. Consequently, it is possible to reduce the initial tube thickness (w) and to save material. Therefore, it is necessary to investigate which is best compromise between initial L and w that allows one to reach H(w_min_) ≥ H_target_ and to use the small initial tube dimensions that are possible.

The objective was therefore to determine the optimal values of L and w that, at the same time, respect the constrain H(w_min_) = H_target_ and minimize the volume of the initial tube.

#### 3.2.1. Numerical Optimization Method—Description

Numerical methods can be used with the aim of optimizing a phenomenon (f) that is a function of an n-dimensional variable $\bar{X}$ and $f\left(\bar{X}\right)$ is not known a priori but it can be estimated in the $\bar{X}$ domain (Dn). If the values that $\bar{X}$ can assume are limited (i.e., Dn is limited) we talk about constrained optimization. Under these conditions, numerical methods allow one to start from an arbitrary point ${\bar{X}}_{0}$ ∈ Dn, to map the trend of $f\left(\bar{X}\right)$ around ${\bar{X}}_{0}$ and to accordingly move a step to a new point $\bar{X}$1 ∈ Dn, which will be closer the next local minimum (or maximum) $\bar{X}$opt ∈ Dn. The procedure is iterated until the current point $\bar{X}$k ∈ Dn is sufficiently close to $\bar{X}$opt (convergence criterion). Since $\bar{X}$k is a local minimum (maximum) for $f\left(\bar{X}\right)$, it is necessary to iterate the algorithm using different ${\bar{X}}_{0}$ values. By comparison, the best of the best amongst the minima (maxima) is accepted as the optimum for $f\left(\bar{X}\right)$ [14,15]. The described method was implemented in Matlab code as follows:

Domain (*n* = 2):

- $\bar{X}$ = (L, w),
- Constrain: L∈(L_min_; L_max_) ∧ w∈(w_min_; w_max_).

Approaching steps:

- Line–search method,
- Step direction: along $-\nabla f\left({\bar{X}}_{i}\right)$,
- Step length (ΔLi, Δwi): 1/10 of the domain amplitude.

Steps close to the optimum:

- Bisection method,
- Step direction: along $-\nabla f\left({\bar{X}}_{i}\right)$,
- Step length (ΔLi, Δwi): ΔLi (or Δwi) halved at each inversion of sign for $\frac{\partial f}{\partial L}$ (or $\frac{\partial f}{\partial w}$),
- Convergence criterion: |ΔLi| ≤ 1 mm ∧ |Δwi| ≤ 0.02 mm.

The function $f\left(\bar{X}\right)$ was defined as a weighted sum of three terms (1): volume of the tube (V), quadratic distance between H(w_min_) and H_target_ and a dummy variable (Fail = (0; 1)), which takes into account the presence of defects on the formed part (i.e., wrinkle or bursting). Weights ($\alpha_{1}$, $\alpha_{2}$ and $\alpha_{3}$) were used to normalize the results when approaching the optimum. In particular $\alpha_{1}$ and $\alpha_{2}$ scale around 1 the terms related to volume and height, while $\alpha_{3}$ scales the fail term at 1000 and it numerically represents a discontinuous vertical wall for $f\left(\bar{X}\right)$. Given its formulation, the optimum of the process corresponds to the minimum for $f\left(\bar{X}\right)$.

(1) $$f=\;\alpha_{1}\times V+\alpha_{2}\times {\left(H\left(w_{min}\right)-H_{target}\right)}^{2}+\alpha_{3}\times Fail$$

(2) $$\alpha_{1}=\frac{1}{V_{\mathrm{avg}}}\;\mathrm{where}:\;V_{\mathrm{avg}}\;=\;\mathrm{volume}\;\mathrm{at}\;\mathrm{the}\;\mathrm{center}\;\mathrm{of}\;\mathrm{the}\;\mathrm{domain}$$

(3) $$\alpha_{2}=\frac{100}{H_{target}}$$

(4) $$\alpha_{3}\;=\;1000$$

**Figure 6:**
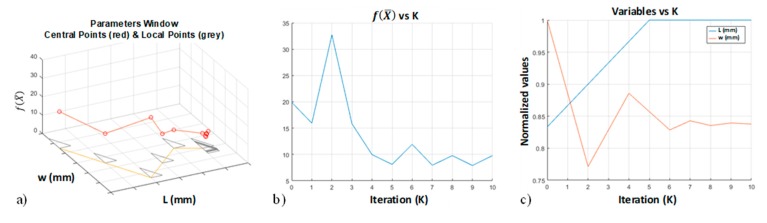
Example of the steps of the optimization algorithm (Large-Tee). In particular, (**a**) 3D representation of $f\left(\bar{X}\right)$ in (L, w) domain; (**b**) values of L and w variables and (**c**) values of $f\left(\bar{X}\right)$ during the algorithm iteration.

The algorithm was implemented using the FEM model previously described. A 3D representation of the optimization algorithm is reported in Figure 6a where the path (yellow line) in the (L, w) domain is reported together with the points (vertex of the grey triangles) used to map the objective function $f\left(\bar{X}\right)$ (red line with circles). The values of $f\left(\bar{X}\right)$, L and w during the algorithm iterations are reported in Figure 6b,c respectively. In particular, from iteration k = 5 the L variable reached its upper constraints becoming stable while w variable converged to its local optimum value. This was reflected by $f\left(\bar{X}\right),$ which fluctuated around its minimum from k = 5.

#### 3.2.2. Numerical Optimization Method—Implementation

**Figure 7:**
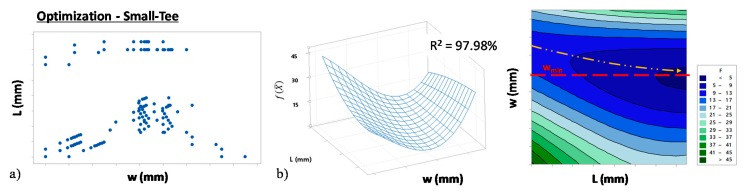
Results of the numerical optimization (Small-Tee). (**a**) Points tested by the algorithm in the (L, w) domain and (**b**) numerical fit of the objective function $f\left(\bar{X}\right)$.

**Figure 8:**
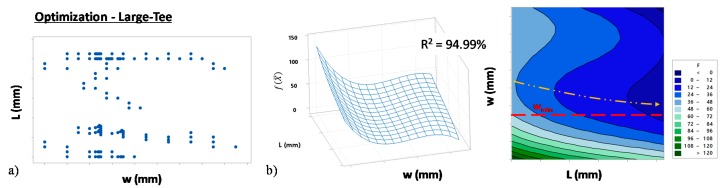
Results of the numerical optimization (Large-Tee). (**a**) Points tested by the algorithm in the (L, w) domain and (**b**) numerical fit of the objective function $f\left(\bar{X}\right)$.

The algorithm was implemented using different starting points ${\bar{X}}_{0}$ for both the tubes geometries (Figure 7 and Figure 8). In particular, Figure 7a and Figure 8a represent the couple of L and w values that were tested during the algorithm iterations for the Small- and Large-Tee respectively. Figure 7b and Figure 8b represent the fitted surfaces of the objective function $f\left(\bar{X}\right)$ for the two parts. In particular, the two surfaces show that $f\left(\bar{X}\right)$ depends on both tube initial thickness (w) and length (L). Moreover, both surfaces show a valley across intermediate values of w and the bottom-valley gradually decreases as L increases. In particular, it is possible to observe that the minimum of $f\left(\bar{X}\right)$ tended to wmin for high values of L. Moreover, the minimum of the function was outside the tested domain. Accordingly, the optimum blank tube that minimized the material cost of the part and allowed us to accomplish the part requirements had an initial thickness close to w_min_. Moreover, its optimum length was outside the tested domain. Overall, this step allowed us to identify the optimal thickness of the initial tube so leaving one last variable to optimize, i.e., the initial length (L). Being the process close to its optimum, the last step was carried out experimentally.

### 3.3. Experimental Tests and Final Adjustments

The previous steps allowed us to identify the process parameters curves (Table 2) and the optimum thickness of the blank tube w = w_min_. Moreover, they indicated the direction for the optimization of the tube length, which has to be higher than those tested so far. Therefore, it was decided to experimentally test the results at the company production plant. On site, slight adjustments on the process curves were introduced to meet the hydroforming machine specifications and to further enhance the process feasibility. The details of the final process configuration cannot be disclosed for confidentiality reasons.

**Figure 9:**
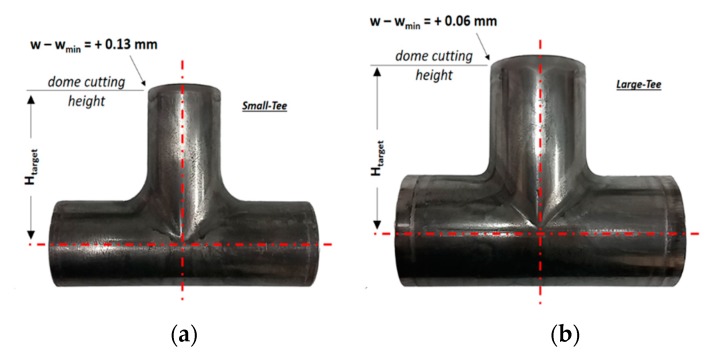
Hydroformed T-joints fabricated using the optimized process curves. (**a**) Small-Tee and (**b**) Large-Tee pieces.

According to the results of the optimization, a blank tube with a thickness w = w_min_ and different lengths were tested until the desired bulge height H = H_target_ was reached (Figure 9). Then, the bulged dome was cut and the thickness of the third branch was measured. Results confirmed that the minimum thickness of the final part was located on the top of the third branch. Moreover, it was close to, and higher than, the minimum value required. In particular, it exceeded the target w_min_ of 0.13 mm and 0.06 mm for the Small- and Large-Tee respectively.

## 4. Discussion

As final step of the developed procedure, the following information could be drawn on the hydroforming process of T-Joints in AISI 316L stainless steel.

Feasibility:

- H(w_min_) = H_target_. It represents the most restrictive geometric requirement to focus on for the design of the process. Moreover, its achievement was very expensive in terms of blank material. In fact, a lot of material accumulated on the feeding sides during the process and only few materials reached the bulged branch of the part increasing H(w_min_).

Significant process aspects:

- Blank Tube Thickness (w). The numerical optimization showed that the optimum thickness of the initial blank tube was close to the minimum thickness allowed on the final part w = w_min_.
- Blank tube Length (L). The length of the initial blank tube had a direct influence on H(w_min_). However, the material accumulated along the feeding sides rather than reaching the expansion zone. Therefore, increasing L to enhance H(w_min_) was not very efficient considering the need of raw material.
- Initial deformation. The deformation that occurred at the beginning of the process was crucial for the minimum thickness of the part. In particular, the minimum thickness in the free expansion area was mainly determined by the deformation of the tube during the initial phases. Therefore, it was important to improve these phases (see the following discussion on precrash and pressure). This result is opposite to what happens in the filling of closed die corners [23] where the minimum thickness is reached at the end of the forming process.
- Precrash. The use of a bulged counter-punch to preform the tube before expansion allowed us to reduce the thinning of the part so increasing its feasibility in accordance to [22].
- Pressure. A low pressure should be adopted in the early phase of the process so expanding the tube and reducing its thinning. After that, the pressure was less influent on thinning and it could be raised to contrast wrinkles. Moreover, as discussed in [24] the pressure is not expected to significantly enhance the height of the bulged branch, which is, as discussed, the most restrictive geometric requirement of the part.
- Other parameters. Process parameters as counter-punch force and punch velocity did not influence the feasibility of the part.

## 5. Conclusions

Tube Hydroforming is a good and effective technology in the pipe manufacturing industry. In fact, it allows one to reduce the number of operation (forming and welding) and the overall time required to manufacture complex parts. Moreover, hydroformed components are more valuable since they are seamless and more resistant due to material hardening. Therefore, the changeover of a production technology towards Tube Hydroforming is of interests for the industry. On the other hand, the change implies feasibility and process design issues, which are complicated by the large number of variables that are usually involved and that need to be optimized.

This paper presented an actual study on the hydroforming process design of AISI 316L stainless steel T-Joints. In particular, it describes the methodology that was adopted to initially evaluate the new production critical features of the part. Furthermore, the degree of freedom in the process design was gradually reduced to simplify the approach. Initially, the optimal process parameter curves were identified. Then, the focus was set on the optimization of the amount of material required to manufacture the part (blank tube dimensions) so to reduce its cost. Being involved more input variables as the tube length and thickness and more output results (volume, part requirements and process feasibility), a comprehensive objective function was defined and optimized. The optimization was performed by implementing two numerical methods, the line–search method to approach the optimum followed by the bisection method for the optimum identification. Results allowed us to identify the best thickness of the blank tube. The last variable to be identified, the length of the blank tube, was experimentally optimized.

The method was implemented on two sizes of Tee-Joints, small and large, and it led to the optimization of the process for both components. Moreover, results show the capability of the procedure that can be generally used to support the design of tube hydroforming processes.

## Figures and Tables

**Table 1:** FEM model details.

**Solver**	PamStamp2G—Explicit
**Meshing**	Die/Punches: Rigid Shell Tube: Deformable Shell–Double symmetry planes
**Material**	AISI 316L—σ = 1453 × (ε + 0.159)^0.634^
**Friction**	Coulomb—Tube–Die: 0.057 Coulomb—Tube–Punch: 0.12

**Table 2:** Process parameter curves optimized and the FEM stability domain.

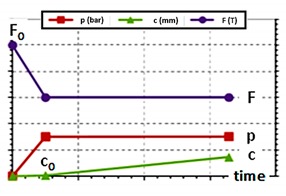		**Small-Tee**	**Large-Tee**
	**Process**		
	Pressure (p)	p = 500 bar	p = 300 bar
	Punch velocity (v)	v_0_ = 1 mm/s v = 5 mm/s	v_0_ = 1 mm/s v = 5 mm/s
	Counter-punch (bulged) force (F)	F_0_ = 2.5 T ^1^ F = 1.5 T	F_0_ = 5 T ^1^ F = 3 T
	**Domain for Tube optimization**		
	Length (mm)	L ∈ (L_1_; L_1_ + 80)	L ∈ (L_2_; L_2_ + 100)
	Thickness (mm)	w ∈ (w_1_; w_1_ + 1.25)	w ∈ (w_2_; w_2_ + 2.00)

^1^ (precrash max 4 mm).
